# How [^18^F]FDG-PET/CT Affects the Management of Patients with Differentiated Thyroid Carcinoma in Clinical Routines

**DOI:** 10.3390/cancers16030588

**Published:** 2024-01-30

**Authors:** Jonas Vogel, Julia Sekler, Brigitte Gückel, Christina Pfannenberg, Konstantin Nikolaou, Christian La Fougère, Helmut Dittmann, Christian Philipp Reinert

**Affiliations:** 1 Nuclear Medicine and Clinical Molecular Imaging, Department of Radiology, University Hospital of Tuebingen, Otfried-Mueller-Strasse 14, 72076 Tuebingen, Germany; 2 Diagnostic and Interventional Radiology, Department of Radiology, University Hospital of Tuebingen, Hoppe-Seyler-Str. 3, 72076 Tuebingen, Germanychristian.reinert@med.uni-tuebingen.de (C.P.R.); 3 Cluster of Excellence iFIT (EXC 2180) “Image Guided and Functionally Instructed Tumor Therapies”, University of Tuebingen, 72076 Tuebingen, Germany; 4 German Cancer Consortium (DKTK), Partner Site Tuebingen, 72074 Tuebingen, Germany

**Keywords:** thyroid cancer, FDG, PET/CT, oncology, patient management, therapy decisions

## Abstract

**Simple Summary:**

[^18^F]FDG-PET/CT is a new imaging modality used in routine practice for many tumor diseases. The combination of morphological and metabolic information has demonstrated superiority over pure morphological imaging, significantly impacting treatment decisions and patient outcomes in many tumor entities. However, the role of [^18^F]FDG-PET/CT in differentiated thyroid carcinoma remains unclear. While potential benefits are acknowledged, there is a lack of clear recommendations and guidelines on when to use this imaging technique. Our study aims to evaluate the influence of FDG-PET/CT on decision making in clinical routines. The impact on clinical decision making was evaluated through questionnaires given to referring physicians before and after PET/CT examinations. The results demonstrate a significant influence of [^18^F]FDG-PET/CT on patient management in our cohort, leading to the avoidance of both additional invasive and imaging diagnostics. Therefore, our study suggests that the (clinically indicated) use of PET/CT in differentiated thyroid carcinoma could result in better therapy management and ultimately lead to improved patient outcomes.

**Abstract:**

Purpose: To investigate the impact of [^18^F]FDG-PET/CT on the management of differentiated thyroid carcinoma (DTC) in routine clinical settings. Material and methods: In total, 98 patients (55 females, age 56 ± 18 years) with histologically confirmed thyroid cancer, including all types of DTC and poorly differentiated thyroid cancer (PDTC, n = 7), underwent [^18^F]FDG-PET/CT for staging or recurrence diagnostics performed using a state-of-the art clinical scanner (Biograph mCT, Siemens Healthineers) with a standardized examination protocol. The impact of PET/CT on clinical decision making was prospectively evaluated using standardized questionnaires completed by the referring physicians before and after PET/CT. Patient outcome was analyzed for OS drawn from patient records. Results: Referring physicians were unable to establish a treatment plan for 81% of patients with thyroid cancer in the absence of PET/CT. The use of PET/CT had a notable influence on patient management, leading to the development of a well-defined treatment plan for 92% of patients. Moreover, after PET/CT a change in pre-PET/CT-intended treatments occurred in 32% of cases, and further invasive diagnostic could be waived in 7% of cases. [^18^F]FDG-PET/CT revealed a tumor detection rate of 68% (local tumor: 19%, lymph node metastases: 40%, distant metastases: 42%). HTg levels, when stimulated via TSH, were considerably higher in patients with metastases detected on PET/CT, compared to those without metastatic findings (*p* = 0.02). OS was significantly worse in patients with PDTC (*p* = 0.002) compared to follicular thyroid cancer (FTC) and PTC or even in patients with distant metastases at first diagnosis (*p* = 0.03). Conclusions: This prospective registry study confirms that [^18^F]FDG-PET/CT used in a routine clinical setting has a very important impact on the management of patients with thyroid cancer by initiating treatments and reducing the uses of additional imaging and invasive tests.

## 1. Introduction

Thyroid cancer represents 3% of all tumor diagnoses with a total amount of approximately 586,000 cases per year worldwide [1], with differentiated thyroid carcinoma (DTC), including papillary (PTC) and follicular thyroid cancer (FTC), accounting for 90% of all cases. Other rare subtypes of thyroid cancer are Hurthle cell carcinoma/oncocytic thyroid carcinoma (HTC), which is classified as a follicular neoplasm with more than 75% oncocytic tumor cells and closely related to FTC [2] as well as poorly differentiated thyroid cancer (PDTC), a neoplasm with heterogeneous diagnostic criteria and intermediate clinical behavior between well-differentiated and undifferentiated thyroid carcinoma [3]. DTC usually comes with an excellent prognosis, and there are numerous guidelines for therapy and follow-up [4,5,6]. There is no standardized treatment for PDTC, but treatment decisions in routine clinical practice are mainly based on experience with treating DTC [7].

Complete thyroidectomy with or without cervical lymph node dissection depending on metastatic risk [8] is recommended as the standard treatment for DTC, including PTC, FTC, and HTC, and therefore, in routine clinical practice, also for PDTC. Lobectomy may be considered sufficient in cases of low-risk DTC [4,9].

According to a consensus recommendation of the European Thyroid Association (ETA), post-surgery risk-based selections of candidates for post-operative radioiodine therapy (RIT) [6] should be performed. Several studies have shown that RIT reduces recurrence and mortality rates [10,11,12]. In case of a palliative situation resulting from radioiodine resistance in an inoperable setting, the symptomatic irradiation of metastasis as well as systemic treatment with tyrosine kinase inhibitors (TKI), like Lenvatinib and Sorafenib [13], may be used.

The potential benefit of using [^18^F]FDG-PET/CT in the staging of thyroid cancer due to the inverse relationship between the uptake of radioiodine and the uptake of [^18^F]FDG, the so called Flipflop phenomenon, has been known for decades [14,15,16]. [^18^F]FDG-PET/CT has been shown to be a valuable imaging modality in well-defined situations, such as with DTC with elevated hTg levels and negative ^131^I whole-body scintigraphy (WBS) [16,17]. In this setting, a prognostic value of the PET parameters, total MTV, and total TGL could be demonstrated [18]. There are also some studies that have investigated the clinical impact of [^18^F]FDG-PET/CT on patient management [18,19], for instance, in the dose adjustment of or exclusion from further RIT or in improved surgical planning. On the other hand, there is no definite recommendation for [^18^F]FDG-PET/CT according to the guidelines of the European or American Thyroid Association (ETA or ATA). Piccardo et al. provided a comprehensive overview of the current issues in the use of PET/CT for DTC and mentioned potential timepoints where there was an indication for [^18^F]FDG-PET/CT in clinical practice [20], e.g., as a part of the initial staging in PDTC and HTC. Rivera et al. demonstrated that, particularly in aggressive histological subtypes, there is an increased risk of progressing dedifferentiation with an iodine negativity of metastases [21].

It has been shown that disease progression leads to the development of aggressive phenotypes of thyroid cancer [22], especially when distant metastases are present at the time of initial diagnosis, resulting in a high prevalence of iodine-negative diseases [23].

Nevertheless, there are no evidence-based data or metanalyses and PET/CT not being recommended as a preoperative staging routine regardless of the differentiation and metastatic grade according to the the ATA guideline [4]. 

Also, cross-sectional imaging may be considered as a prognostic tool for patients with metastatic disease or patients at the highest risk for rapid disease progression. Beyond that, PET may be considered for the assessment of the posttreatment response following systemic or local therapy for metastatic or locally invasive diseases, but according to the ATA, whether conventional imaging (MRI and CT) or [^18^F]FDG-PET/CT should be used is still controversial [4]. The ETA guideline states that [^18^F]FDG-PET/CT may yield additional information on tumor biology, but the authors go on to emphasize that it remains to be shown whether this has a prognostic or management impact [5].

The only situation with a strong recommendation for the use of [^18^F]FDG-PET/CT is defined as elevated serum Thyroglobulin (hTg) levels in high-risk DTC patients and negative radioiodine imaging [4,5]. Usually a TSH-stimulated hTg ≤ 10 ng/mL is seen as a cutoff level, but there are also other cutoff-levels discussed in the literature [24], and this level needs to be adapted and lowered in case of aggressive pathological variants of thyroid cancer that may produce only low amounts of serum hTg [4]. Additionally, it has been shown that instead of an absolute level of hTg, the hTg doubling time might be a more sensitive marker for the recommendation of an FDG-PET/CT [25].

In summary, the use of [^18^F]FDG-PET/CT in DTC may be promising in many situations where clear recommendations are lacking. As a result, the use of [^18^F]FDG-PET/CT in clinical routine is likely to vary considerably from center to center. This prompted us to analyze the real-world data prospectively recorded in our PET/CT registry. This study involves all DTC subgroups, including HTC as well as PDTC, and aims to investigate the impact of [^18^F]FDG-PET/CT on the treatment decisions for these patients.

## 2. Materials and Methods

### 2.1. Patient Cohort

The patient cohort studied here was prospectively enrolled and incorporated in our local PET/CT registry between April 2013 and November 2020 and included all patients with DTC, including Hurthle cell variants and poorly differentiated tumor types. Preliminary results including various tumor entities from April 2013 to August 2016 were previously reported in a study by Pfannenberg et al. [26]. In addition, our work represents the first detailed analysis of a patient subgroup with differentiated thyroid carcinoma. Detailed information on patients is compiled in Table 1. For patients who received more than one PET/CT during the study period, only the first scan was evaluated to avoid bias. The follow-up period lasted until February 2022. All PET/CT examinations were requested by the attending physicians, then reviewed by a board-certified nuclear medicine specialist and a specialist in radiology, and reported in consensus. Indications for PET/CTs according to the referring physicians were first treatment (n = 48), restaging (n = 9), and suspected relapse (n = 41). The TNM status was determined according to the AJCC based on available pathological reports at the time of initial diagnosis (1985–2020), utilizing various versions but predominantly the 7th and 8th versions (only 19 patients were diagnosed before 2009).

### 2.2. PET/CT Acquisition

All PET/CT examinations were performed using a state-of-the art clinical scanner (Biograph mCT^®^, Siemens Healthineers, Erlangen, Germany). Before the examination, all patients had to fast for at least 8 h. The PET data acquisition started 60 min after an intravenous application of 300–350 MBq [^18^F]FDG, and data were acquired from the skull base to the mid-thigh level over six to eight bed positions with 2 min acquisition per bed position. Reconstruction was conducted using a 3D ordered-subset expectation–maximization algorithm (two iterations, 21 subsets, Gaussian filter: 2.0 mm, matrix size: 400 × 400, and slice thickness: 2.0 mm). If no radioiodine therapy was planned and other contraindications could be excluded, weight-adapted 90–120 mL intravenous CT contrast agent (Ultravist 370, Bayer Healthcare Pharmaceuticals Berlin, Germany) was injected for CT examination. After the iterative reconstruction of PET images, they were co-registered with CT data by applying commercial software (syngo.via^®^ and True D^®^; Siemens, Erlangen, Germany). In the majority of cases, PET/CT acquisition occurred without TSH stimulation; only 26 patients underwent TSH stimulation, typically for radioiodine imaging and PET/CT at the same time.

### 2.3. Data Analysis

Patient details such as TNM stage, histological tumor type, date of cancer diagnosis, prior therapies, and level of human Thyroglobulin (hTg) at the time of PET/CT and during follow-up were recorded from in-house clinical reports. In the case of TSH-stimulated hTg values, differentiation between exogenous and endogenous TSH stimulation was not performed.

Intended patient management was assessed both prior to PET/CT and in knowledge of the PET/CT findings using standardized questionnaires completed by the referring physicians as part of the registry study and cross-referenced with clinical reports. The questionnaires were designed based on the conception of the “National Oncologic PET Registry” (NOPR) which is described in detail in the publications of Hillner et al. and Pfannenberg et al. [26,27].

PET/CT findings were extracted from clinical consensus reports. Follow-up (death or last vital status) was assessed for all patients based on available patient records.

### 2.4. Statistical Analysis

Statistical analyses and graphical illustrations were performed using JMP^®^ software (Version 13.1, SAS Institute Corporation, Heidelberg, Germany) and an open-source software (SankeyMATIC^®^). The Gaussian distribution of continuous variables was tested using the Kolmogorov–Smirnov test. Differences in overall survival between various subgroups were analyzed using non-parametric log-rank tests, differences regarding other variables were evaluated using a two-sided *t*-test. The significance level was set at a *p*-value of <0.05; Bonferroni correction was employed to counteract the problem of multiple comparisons and correct the local alpha-level. The median survival time was derived via Kaplan–Meyer analysis. 

## 3. Results

### 3.1. Patient Cohort

A total of 98 patients (55 females, average age 56 ± 18 years) were analyzed. Thyroid carcinoma was histologically confirmed in all patients: 57 papillary, 28 follicular, 4 Hurthle cell, 7 poorly differentiated, and 2 mixed papillary/follicular carcinomas. Because of their close association, HTC and FTC were combined for further analysis, and mixed papillary/follicular carcinomas were also included in the FTC/HTC group. Detailed patient information is provided in Table 1.

Clinical indications for PET/CT could be subgrouped as follows: first treatment (n = 48), imaging in case of suspected relapse (n = 41), and restaging for therapy control (n = 9); patient characteristics for subgroups are also summarized in Table 1. As expected, in the group of patients undergoing first treatment, the median age (*p* < 0.01), hTg levels (suppressed TSH; *p* = 0.05) as well as average dose of previous radioiodine treatment (*p* = 0.01) were lower compared to those of the total cohort. Besides this, no difference between the subgroups could be found.

**Figure 1:**
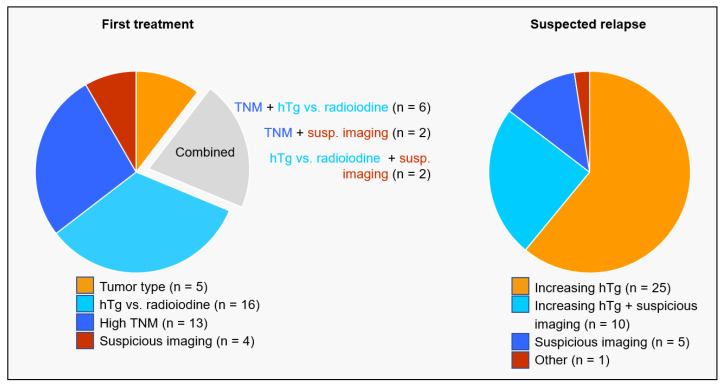
Reasons for PET/CT evaluation. PET/CT examination as part of the initial staging (n = 48) was based on aggressive tumor type, mismatch between hTg level and radioiodine imaging as well as an inadequate decline in hTg after radioiodine treatment (hTg vs. radioiodine), a high TNM stage, or suspicious image findings. PET/CT in case of suspected relapse (n = 41) was caused by an increasing hTg level or suspicious imaging finding. In one case, PET/CT was performed in a patient with a high initial TNM and with autoantibodies against hTg.

Reasons leading to a PET/CT examination at initial staging as recorded by referring physicians were aggressive tumor type (poorly differentiated thyroid carcinoma), a high TNM stage (distant metastases, M1, or lymph node-positive, locally advanced tumor stage, T4 N+), image findings suspicious for iodine-negative tumor prior or during radioiodine treatment, or mismatch between hTg level and radioiodine imaging as well as an inadequate decline in hTg after radioiodine treatment; see also Figure 1. 

PET/CT indication in case of suspected relapse was based on an increasing hTg level or suspicious imaging findings in conventional cross-section imaging or cervical ultrasound. In one case, PET/CT was performed in a patient with a high initial TNM and with autoantibodies against hTg, preventing the evaluation of the hTg level in this patient; see also Figure 1.

Due to the small subgroup, “restaging for therapy control” was not evaluated further.

### 3.2. PET/CT Findings

Overall, PET/CT resulted in a tumor detection rate of 68% (n = 67). Local tumors were detected in 19 patients, and lymph node metastases in 40 patients; 38 patients had distant metastases, with lung (n = 30) and bone (n = 12) being the predominate metastatic sites. In addition, liver or brain metastases were found in two patients and pancreatic metastasis in one patient. No histological confirmation of pancreatic metastasis was performed, as the lesion characteristics (supplementary pancreatic MRI conducted), the distribution pattern, and the metabolic behavior of the metastasis excluded a pancreatic second neoplasm with high certainty. Furthermore, the subsequent course (>4 years of follow-up) confirmed the suspected diagnosis of pancreatic metastasis from thyroid carcinoma. There was no significant difference in tumor detection rate or detection site between the first treatment group and imaging in case of suspected relapse group; see Table 1.

Our results demonstrate significantly higher hTg levels under TSH stimulation (TSH > 30 IU/L) when PET/CT showed evidence of metastasis than that in in metastatic-free patients (*p* = 0.02). A similar trend was shown for hTg levels in patients with TSH suppression (TSH < 0.5 IU/L), although not reaching significance, possibly due to the smaller number of patients.

There were also similar trends toward higher rates of metastases in patients with FTC/HTC and PDTC compared with PTC, as well as in high-risk patients (according to the ATA), although none of these trends were significant. 

PET/CT scanning yielded some relevant additional findings: breast cancer (n = 1), renal cell carcinoma (n = 1), and metastatic prostate carcinoma (n = 1), as well as severe pneumonia treated with pleural drainage (n = 1). All tumor diagnoses were histologically confirmed.

### 3.3. Management Changes in Therapy Planning Due to PET/CT

**Figure 2:**
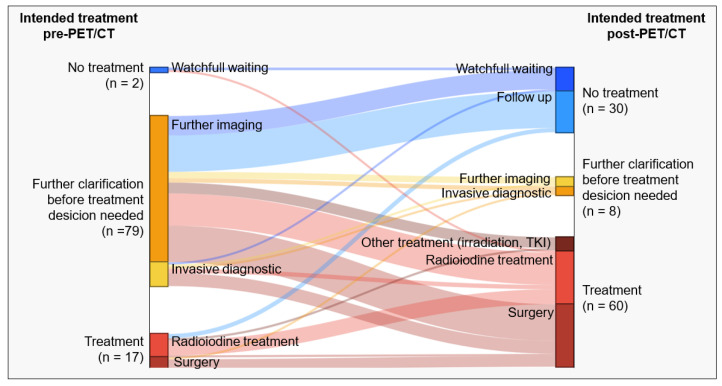
Changes in treatment concept due to PET/CT findings. Overview of the influence of PET/CT on planned patient treatment: The left side depicts the treatment plan before PET/CT, while the right side shows the plan after PET/CT. Colored bars and their strength represent the flow of patients between treatment groups. Pre-PET/CT, there were few cases with a defined treatment plan (watchful waiting: n = 2, radioiodine treatment: n = 11, surgery: n = 6). Most cases required further clarification via imaging (n = 68) or invasive diagnostics (n = 11). PET/CT results prompted referring physicians to change patient management in many cases, allowing the definition of treatment strategies (watchful waiting: n = 10, follow-up: n = 20, radioiodine treatment: n = 24, surgery: n = 27, other or combined treatment: n = 9) with only 8 cases needing further clarification. Additionally, in 32% of cases with an intended treatment plan before PET/CT, adjustments were made, including significant changes for three patients (from no treatment to treatment: n = 1, or treatment to no treatment: n = 2).

The clinical management of thyroid cancer patients has been significantly influenced by additional PET/CT imaging. Changes in treatment concept based on PET/CT findings are shown in Figure 2. Prior to PET/CT, referring physicians regarded further clarification before definite treatment decision necessary in 79 cases (80%), recommending cross-sectional imaging (MRT or CT, n = 68) or invasive diagnostic procedures (i.e., biopsy, panendoscopy, n = 11). Without PET/CT, a treatment plan was prepared in only a few cases (n = 19, radioiodine treatment: n = 11, surgical treatment: n = 6, no treatment/watchful waiting: n = 2), which in 6 cases, also had to be adjusted in response to the PET/CT findings. 

Taking the PET/CT results into account, further diagnostic workup was required in only 8% of cases, with either a decision for additional imaging (n = 4) or invasive diagnostics (n = 4).

According to referring physicians and patient records, a treatment planned with knowledge of PET/CT was surgery alone (n = 26 cervical, n = 1 pulmonary), radioiodine treatment alone (n = 24), systemic treatment with TKI (n = 5, cervical radiotherapy (n = 1), and combined treatment for 3 patients (n = 2 combined cervical surgery and RIT, n = 1 combined surgery and radiotherapy of bone metastases). Further follow-up without treatment was recommended for 20 patients who had no evidence of a tumor in PET/CT. In 10 other patients, treatment was not considered useful at the time because of an unfavorable balance between treatment benefit and potential adverse effects, typically in cases of slow tumor progression. Instead, watchful waiting was preferred. There was no significant difference in the change in treatment strategy between the first treatment subgroup and patients with suspected relapse.

With the exception of six treatments (n = 4 surgical procedures, n = 2 TKI treatments), all therapies were performed at our center. In the majority of cases (52/56), the planned treatment was subsequently carried out: only in two cases, the planned surgery; and in one case, the radioiodine treatment was not realized. In another case, further therapy remained unclear, as the patient was lost at follow-up.

**Figure 3:**
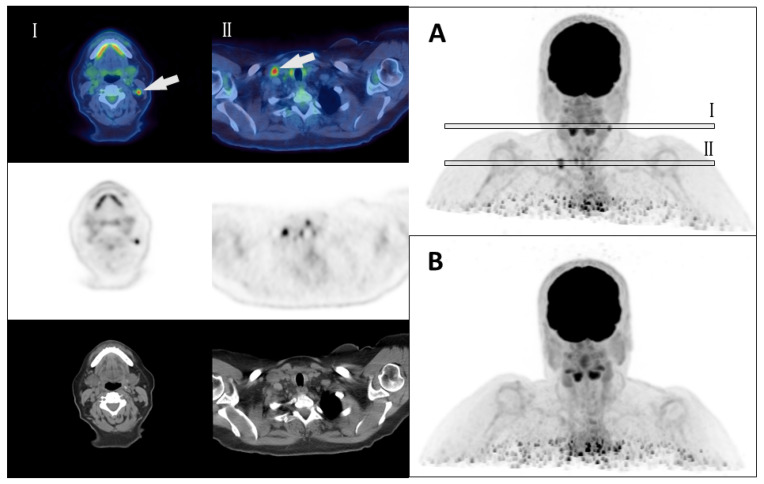
Example of pre- and post-therapeutic [^18^F]FDG-PET/CT findings. Pretherapeutic [^18^F]FDG-PET/CT imaging (**A**) shows multiple focal FDG lesions (n = 5) classified as lymph node metastases in a patient with high-risk papillary thyroid cancer (T3, N1, R1) in the 3D-PET-reconstruction. Two of these metastases are marked with a white arrow in cross-sections (**I**) and (**II**). Decision for PET/CT was made due to a mismatch of a high hTg Level of 72.5 ng/mL (endogen TSH stimulation) and only little iodine-avid tissue in the first radioiodine treatment. Following PET/CT, a cervical lymph node dissection was performed confirming multiple lymph node metastases (n = 6). A second [^18^F]FDG-PET/CT imaging after surgery demonstrated the absence of any FDG-avid lesions (**B**) and hTg dropped below the detection limit until the end of the follow-up period (73 month from resection).

In the case of surgery, tumor was detected in the resected tissue in 24 of 28 cases (86%), indicating true-positive PET/CT findings. Examples of pre- and postsurgical PET/CT finding are shown in Figure 3. No tumor tissue was found in only two cases, and histological data were not available in two cases. Surgery resulted in a decrease in hTg in 22 of 28 cases (79%), with 10 patients (36%) achieving an hTg below the detection limit (<0.5 µg/mL) after surgery. It should be noted that even in one patient in whom no tumor tissue was detected in the resected specimen, there was a decrease in their postoperative hTg level. No drop in hTg level was observed in only three cases. For three patients, no information on hTg levels was available.

With RIT alone, a post-therapeutic drop in hTg occurred in 14 of 24 patients (58%), with hTg turning negative (<0.5 µg/mL) in 7 cases (29%). No drop in hTg was observed in only six cases, while no data on the pre- or post-therapeutic hTg were available for four patients.

Patients who underwent TKI treatment responded in two cases, while one patient died immediately after starting TKI treatment. No further information was available for two other patients also planned for TKI.

The patient who received cervical radiation alone had stable disease locally but developed progressive pulmonary metastases.

In the case of a pre-PET/CT-planned therapeutic concept (radioiodine therapy n = 11, surgery n = 6, watchful waiting n = 2), there was a modification of the therapeutic approach in 32% (n = 6) of cases based on the results of the FDG-PET/CT examination. This resulted in one case of therapy escalation (change: watchful waiting to radioiodine treatment) and in two cases of de-escalation (further radioiodine treatment to follow-up). The remaining three cases involved transitions between therapeutic modalities (radioiodine therapy to surgery or TKI) with one case requiring a preceding clarification through biopsy.

### 3.4. Outcome

The follow-up period was 55.9 ± 30.4 months (minimum 14.3 months). During this time, there were 10 tumor-related and 6 non-tumor-related deaths; thus, the median OS was not reached in this study. Regarding the six non-tumor-associated deaths, four were likely due to age-related causes in elderly patients with a stable tumor status at the time of death (69, 74, 79, and 86 years). One patient died from a non-metastasis-related cerebral hemorrhage, and another patient due to the consequences of a metastasized small cell lung carcinoma (initial diagnosis after PET/CT imaging). Among the ten tumor-related deaths, tumor type was follicular/oncocytic carcinoma in five, poorly differentiated carcinoma in two, and papillary thyroid carcinoma in three cases. In the PET/CT, distant metastases were detected in 8 out of 10 patients, with no tumor evidence in the PET/CT for only 2 patients. 

Of the 82 patients who were alive at the end of the follow-up period, 73 patients (89%) were followed-up with regularly (at least once a year) at our center. There was a trend toward shorter OS in patients with metastatic disease detected via PET/CT, especially for the subgroup with distant metastases.

**Figure 4:**
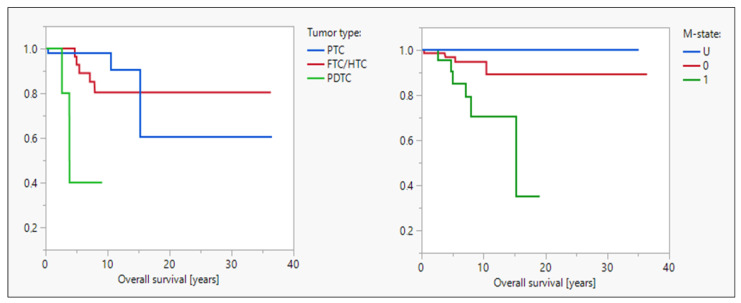
Impact on OS. OS (since first tumor diagnosis) was significantly shorter in patients with PDTC (median OS, 3.9 years) compared to those with FTC/HTC and PTC (median not reached, *p* = 0.002). Patients with distant metastases at initial diagnosis also had significantly shorter OSs (median OS, 15.2 years) compared to patients without distant metastases or with an unknown metastatic status (*p* = 0.03).

Patients with PDTC had a significantly shorter OS since first tumor diagnosis (median OS, 3.9 years, *p* = 0.002) compared to those with FTC/HTC and PTC. A similar difference was seen for patients with distant metastases at first diagnosis (median OS, 15.2 years, *p* = 0.03); see also Figure 4. There was also a trend for differences in OS in the dependence of risk classification according to the ATA, but this was not significant probably due to unequal distribution within the groups.

## 4. Discussion

Progressive metastasis in well-differentiated thyroid carcinomas and especially in already primary aggressive tumor types or initial distant metastasis may lead to increased differentiation with mixed iodine affinity [21,22,23]. In such cases, there could be a presumed benefit from using FDG-PET/CT in staging patients and subsequent treatment planning. The aim of this study was to prospectively evaluate the impact of clinically indicated [^18^F]FDG-PET/CT imaging on the management of patients with all subgroups of DTC, including HTC and PDTC, in a real-world setting.

Meanwhile, there is a substantial data foundation demonstrating that FDG-PET/CT exhibits increased sensitivity and specificity in staging various tumor diseases compared to conventional cross-sectional imaging [28]. Additionally, numerous, partially randomized controlled studies have been conducted for various tumor entities, highlighting the positive impact of FDG-PET/CT on clinical decision making [29,30,31,32]. Two large PET/CT registry studies, summarizing the results of these individual studies, also contribute to this evidence [26,33]. Aligning with the presented results, we observed a modification in the predetermined therapeutic concept in 32% of cases in our cohort, closely resembling the reported therapy changes of 36.5% [33] and 37.1% [26]. It is worth noting that the proportion of patients without a clearly defined therapeutic concept before FDG-PET/CT imaging in our cohort with 78% is significantly higher than the 42% reported by Pfannenberg et al. [26], highlighting the substantial clinical influence of PET/CT, particularly in the examined tumor entity of DTC.

On the other hand, there have been only a few studies on patients suffering from DTC that have investigated the clinical impact of [^18^F]FDG-PET/CT on patient management [18,19], such as dose adjustment or the exclusion of further RIT. Larg et al. showed that [^18^F]FDG-PET/CT can influence clinical decision making for DTC with elevated hTg levels and a negative ^131^I WBS, leading to a change in treatment strategy in 33.5% (58/173) of cases [17]. Filippi et al. demonstrated an impact on clinical patient management in 42.4% of cases in a similar setting (DTC with elevated hTg levels and a negative 131I whole-body scan) [34]. Furthermore, it has been demonstrated that certain PET/CT parameters (total MTV and total TLG) possess a prognostic predictive value under specific conditions [35]. However, a common limitation of almost all of these studies [17,18,19] is that they included only a small, well-defined subset of patients and therefore may not reflect the presumed clinical routine in many hospitals.

In contrast, the current study included a comprehensive cohort of DTC patients undergoing [^18^F]FDG-PET/CT based on an interdisciplinary decision and documented regular follow-up at the study center. The major goal of the study was to evaluate whether [^18^F]FDG-PET/CT has a clinically relevant impact on the treatment decisions made by the referring physicians of these patients. In our cohort, only 19% of patients had an established treatment plan prior to PET/CT. With knowledge of the PET/CT results, this rate was increased to 92%. According to the referring physicians, 67% of all patients required further cross-sectional imaging if [^18^F]FDG-PET/CT was not available, which could be reduced to 4% after PET/CT, which means that [^18^F]FDG-PET/CT replaced other cross-sectional imaging in most cases, thus reducing negative impacts on patient comfort. In addition, PET/CT could have avoided invasive diagnostics and planned (radioiodine) treatment in 10 and 2 cases, respectively, representing a significant advantage in patient care. Additionally, by avoiding such non-necessary (invasive) diagnostics and therapies, there can also be a benefit in terms of healthcare costs through FDG-PET/CT.

In 32% of patients, the existing treatment plan had to be adjusted after PET/CT, which could be expected to result in a better outcome for the patient, although such an assumption is difficult to verify. The intended treatment based on PET/CT was actually performed in most cases (52/56), leading to an hTg level response in many cases, and even showed a complete hTg response in 31% of patients who underwent RIT or surgery.

Survival analysis was not the main objective of this study and was obviously prevented by the low mortality rate despite a comparatively long follow-up of 55.9 ± 30.4 months. As a result, analyses of the survival benefit in the subgroups showed only a trend but no significance. Nevertheless, we confirmed the fact that lung and bone metastases are predominant distant metastatic sites in DTC [36].

A general limitation of studies on decision making, such as this registry study, is sampling bias, potentially leading to an overestimation of PET/CT findings [26,27]. As mentioned by Pfannenberg et al. [26] regarding the total cohort of the PET/CT registry study, we also cannot individually trace the circumstances that led the referring colleagues to choose PET/CT over conventional imaging in the cohort of differentiated thyroid carcinomas. However, the interdisciplinary review of the PET/CT request by a board-certified nuclear medicine specialist and a specialist in radiology emphasizes the clinical relevance on a case-by-case basis. Also, it may be considered beneficial that most patients were closely associated with our center, and therefore, the treatment decision, implementation of therapy, and follow-up are mostly well documented, and we were able to perform a cross-check for sufficient clinical evidence.

The number of patients was limited due to the relatively small number of [^18^F]FDG-PET/CT indications performed in clinical routines. This is due to the well-known favorable course in most cases of DTC. Furthermore, the indication for PET/CT was a clinical decision and not based on predetermined criteria such as TNM stage or hTg cut-offs, which is obviously a bias that cannot be avoided in this setting. One may also argue that the heterogenous composition of our cohort with a wide range of hTg levels, tumor stages, and previous therapies is another limitation of this study, but we would like to point out that we believe this is an advantage rather than a limitation, showing that even without focusing on specific situations, [^18^F]FDG-PET/CT, at least at our center, has an enormous impact on patients suffering from DTC.

## 5. Conclusions

The main finding of this study is that [^18^F]FDG-PET/CT has a significant impact on treatment decisions for patients with thyroid cancer, including DTC (PTC, FTC, and HTC) and PDTC, when the PET/CT indication is based on routine clinical practice. In our cohort, treatment decisions could be made after PET/CT for 92% of all patients, resulting in an hTg level response in many cases and even showing a complete hTg response in 31% of patients who underwent RIT or surgery.

## Figures and Tables

**Table 1:** Patient characteristics (n = 98).

	All Patients (n = 98)	First Treatment (n = 48)	Suspected Relapse (n = 41)	Restaging (n = 9)
Sex	male: 43	male: 24	male: 18	male: 1
	female: 55	female: 24	female: 23	female: 8
Age [years]	56 ± 18	48 ± 18	64 ± 16	60 ± 11
Time since first diagnosis [month]	48 ± 71	6 ± 3	91 ± 86	79 ± 52
hTg level [µg/mL]				
stimulated (TSH > 30 IU/L)	346 ± 1145	187 ± 695	313 ± 722	69 ± 142
	(n = 69)	(n = 42)	(n = 26)	(n = 7)
suppressed (TSH < 0.5 IU/L)	67 ± 360	6 ± 21	104 ± 495	3417 ± 3629
	(n = 56)	(n = 18)	(n = 33)	(n = 3)
Referring physician				
intern	92	47	37	8
extern	6	1	4	1
Tumor type				
papillary	57	32	23	2
follicular/oncocytic	32	11	15	6
mixed papillary/follicular	2	-	1	1
poor differentiated	7	5	2	-
Risk classification (American Thyroid Association)				
unknown	12	-	10	2
low	8	4	3	1
intermediate	23	15	7	1
high	55	29	21	5
Initial cTNM stage				
TU	7	-	6	1
T1	17	9	6	2
T2	19	11	5	3
T3	48	25	20	2
T4	7	3	4	-
NU	4	1	3	-
N0	42	16	21	5
N1	52	31	17	4
MU	3	-	3	-
M0	72	36	32	4
M1	23	12	6	5
Previous treatments				
radioiodine treatment (Dose [GBq])	94 (8.9 ± 7.5)	45 (5.7 ± 2.8)	41 (11.2 ± 8.9)	9 (15.6 ± 9.1)
thyroidectomy ± lymphadenectomy	97	47	42	9
local treatment of distant metastasis (radiation/surgery)	13 (8/5)	3 (2/1)	6(5/1)	4(2/2)
PET/CT findings				
local tumor	19	8	9	1
lymph node metastasis	40	17	20	3
distant metastasis	38	14	16	8
total (any tumor finding)	67	27	31	9

## Data Availability

The datasets used and/or analyzed in the current study and that are not included in this published article are available from the corresponding author upon reasonable request.
